# Post-conflict opponent affiliation reduces victim re-aggression in a family group of captive arctic wolves (*Canis lupus arctos*)

**DOI:** 10.1371/journal.pone.0187450

**Published:** 2017-11-06

**Authors:** Martina Lazzaroni, Sarah Marshall-Pescini, Simona Cafazzo

**Affiliations:** 1 Wolf Science Centre, Messerli Research Institute, University of Veterinary Medicine, Vienna, Medical University of Vienna, University of Vienna, Veterinaerplatz 1, Vienna, Austria; 2 Comparative Cognition, Messerli Research Institute, University of Veterinary Medicine, Vienna, Medical University of Vienna, University of Vienna, Veterinaerplatz 1, Vienna, Austria; University of Portsmouth, UNITED KINGDOM

## Abstract

Post-conflict affiliative interactions have been widely investigated in primates but not extensively in other species. Using the Post Conflict-Matched Control (PC-MC) comparison method, this study investigated the patterns of post-conflict opponent affiliation (POA) of a captive family group of 19 arctic wolves (*Canis lupus arctos*), investigating the correlation with various factors. We found that POAs occurred mainly in the non-feeding context and more often when the victim was dominant and the aggressor subordinate. Furthermore, POAs were more likely to have been initiated by the victim than the aggressor. Victims’ stress related behaviours occurred more in PC than MC periods, and more after high vs. low intensity aggressions but they were not more likely to occur after conflicts between wolves with a stronger social bond and POAs did not reduce their rate of occurrence. Our results showed that re-aggression was twice less frequent when a friendly interaction occurred between the aggressor and the victim, and consistent with this, victims engaged in POAs more often than the aggressor. Overall, our results support the hypothesis that POAs in wolves may have been selected for as a mechanism to avoid conflict escalation, which could lead to social disruption and hence jeopardize cooperative activities. The high relatedness among individuals in the pack and the greater dependence of all members on cooperation in breeding and hunting may reduce the importance of ‘relationship quality’ as a mediating factor of POAs, although dominance relationships, which are directly linked to the risks of further conflicts, do play an important role.

## Introduction

Reconciliation is classically defined as the tendency by former opponents of a conflict to contact each other and to engage in affiliative behavioural patterns relatively shortly after a conflict [1]. However, the term 'reconciliation' *per se* implies that these behaviours are a 'consolatory act' [1] (i.e. they reduce the victim’s stress levels) and in species where this function has not been shown the more descriptive term ‘post conflict opponent affiliative interactions’ (POAs), more accurately applies.

Many hypotheses have been suggested to explain why opponents engage in post conflict affiliative interactions, each one focusing mainly on one core aspect i.e. repairing potential damages to the relationship caused by the conflict (*Relationships Repair Hypothesis* [2–8]), restoring valuable relationships (*Valuable Relationship Hypothesis* [9]), reducing the stress caused by the conflict bringing uncertainty in the relationship (*Uncertainty Reduction Hypothesis* [2]). As a sum of the previous hypotheses Aureli [10] outlined the *Integrated Hypothesis*, which has been supported by the most recent studies on primates [11,12]. The Integrated Hypothesis suggests that following a conflict, the former opponents should experience anxiety that reflects both the disturbance effect on their relationship and the risk of renewed attacks. High levels of post-conflict stress would motivate the opponents to seek out POAs as a way to reduce their stress levels, and such behaviour would then serve to repair the disrupted relationship and reduce the risk of further aggressions. Since stress levels are expected to be higher following conflicts between individuals who share a high-quality relationship, post-conflict stress would mediate an increased conciliatory tendency between individuals with good relationships [10].

Although the *Integrated Hypothesis* and the other cited hypotheses have been tested quite extensively in primates, so far only a few studies have investigated their validity in non-primate species (e.g. a review: [13,14]). A broader comparative approach involving a greater variety of different taxa is important since it allows an assessment of a) how widespread POAs are amongst social animals; b) whether hypotheses deriving from the primate literature are valid also outside this taxa or whether POAs may have other functions in different species. Furthermore, an analysis of the social organization characterizing the different species, in which reconciliation is observed, may shed light on the evolutionary factors, which may have influenced the selection for conciliatory behaviours.

Many canids species are highly social, showing strong dependence on cooperative behaviours in the form of cooperative hunting, pair-bonding and communal pup-rearing (for a review of the social organization of Canids see [15]). Reconciliatory behaviours may be particularly important for those species that heavily rely on cooperative activities between group members (e.g. [16–19]). Hence from this perspective, wolves are a particularly interesting study subject, since they are a highly cooperative species (e.g. [20–26]). Wolves live in family groups [27] composed of a long-term breeding pair and their offspring of different generations [27], although unrelated individuals have also been observed joining established packs (e.g. [28]). The females reach the sexual maturity in the second or third year but normally only the alpha female reproduces successfully [29]. The breeding season occurs generally from late autumn or early winter to April depending on the region where wolves live [27]. Offspring may disperse at an age ranging from 9 months to 4.5 years [29] either voluntarily or forced by the other pack members [30]. Wolves strongly rely on cooperation both for hunting large prey [31], territorial defence [26] and pup-rearing (for example by nourishing the pups and the lactating female [20,32]). Hence the cohesiveness and functionality of the packs are crucial for allowing pack members to survive [33,34,35]. Nevertheless, conflicts between pack members do occur as an inevitable consequence of competition over resources observed in group living species [27,36]. In order to mitigate aggression in such species a common social mechanism evolved is the establishment of a dominance hierarchy between pack members [16]. From studies in captivity it has been observed that wolf packs form a linear dominance hierarchy [37–39] influenced by age, where older wolves are dominant on the younger ones [39]. Although the possibility that captivity may have an influence on hierarchical structure has often been mentioned [40,41], in a recent metanalyses of 85 species, Shizuka and McDonald [42] found that results pertaining to the hierarchical organization of a species did not vary in relation to captivity.

Overall, whereas dominance is thought to serve as a mechanism to prevent the occurrence of conflicts between pack members, conflict management strategies are thought to be necessary to restore disrupted relationships when conflicts do in fact occur, thereby maintaining the cohesion of the social group and hence the benefits this provides [3,4,5,7,13,36,43,44].

So far, only two studies have been carried out on POAs in wolves [38,45]. In a family group of captive grey wolves (*Canis lupus*), Cordoni and Palagi [38] found that both victim and aggressor initiated POA; however, neither hierarchical nor affiliative relationships between the opponents affected the likelihood of its occurrence. In a more recent study, the occurrence of POA was found to occur also in a wild population of Canadian timber wolves (*Canis lupus occidentalis*) [45]. In this study, victims were found to initiate post-conflict friendly contacts with their former opponent more often than aggressors. According to authors, POA were directed mostly from subordinates to dominant individuals. However, the latter statement is problematic, since the authors did not explain how they assessed the hierarchical relationship between pack members. In fact, it is not clear whether the hierarchical structure of the wolf pack was independently analysed and it rather seems that the victim was assumed to be subordinate, and the aggressor dominant.

Both studies found that POAs occurred in the study populations, and authors referred to these as reconciliatory acts, even though their function in wolves is still not clear. According to the *Integrated Hypothesis*, the likelihood of re-aggression should decrease after a reconciliatory act but when this was looked at in wolves it was not found to be the case [45]. Moreover, a higher frequency of POAs between opponents that share a good quality relationship is expected. However, so far, this pattern of results has not been detected in wolves [38]. Finally, as predicted by *Integrated Hypothesis*, it has been shown in primates that reconciliation has a stress-reduction function with victims showing a reduced frequency of stress-related behaviours after a POA with the aggressor than in the absence of POAs [2,46–51]. However this hypothesis has yet to be tested in wolves.

In the current study we investigated the existence of post-conflict affiliative behaviours between former opponents in a family group of arctic wolves and the function of these behaviours.

We tested the four main predictions deriving from the *Integrated Hypotheses*:

1. former opponents should seek and make friendly contact at higher rates (and sooner) after a conflict than at other times [6,52,53];
2. victim’s rates of stress related behaviours should be higher after conflicts than in a control situation and higher for individuals that share a high-quality relationship;
3. POAs should be more likely to occur between former opponents that share a good quality relationship [13,54];
4. reconciled conflicts should decrease the likelihood of re-aggression and potentially reduce the rate of stress-related behaviours [10,13,54].

Furthermore, to allow a comparison with previous studies with wolves, we also investigated: 1) how sex composition of the dyads and the rank relationship influence the occurrence of POAs; 2) who is more likely to initiate the POA (victim vs. aggressor) and 3) how the intensity of conflict (high: involving physical contact vs. low: no contact, threat behaviour) and the presence/absence of food (context) may affect the occurrence of POAs. Finally, previous studies on other species found a lower rate of POAs during the mating season [55,56]. Since wolves are seasonal breeders (with mating occurring once a year), we also tested the effect of the breeding season on the occurrence of POAs.

## Materials and method

### Ethics statement

The study was purely observational with no manipulation of animals. The relevant committee, Tierversuchs-kommission am Bundesministerium für Wissenschaft und Forschung (Austria) and all institutions involved (Wolf Science Center, Messerli Research Institute, University of Veterinary Medicine of Vienna, and Olomouc Zoo) allow us to run this research without special permissions regarding animals (wolves) since this is not required for such observational studies (Tierversuchsgesetz 2012– TVG 2012). Permission to observe and video record the wolves’ behaviour was obtained from Olomouc Zoo.

### Subjects and study site

The subject of this study was a pack of captive arctic wolves (*C*. *lupus arctos*) hosted at the Olomouc Zoo (Moravia, Czech Republic). The pack was structured as a family group with all members born into the pack except for the breeding male and the two unrelated breeding females, that arrived in the zoo in 2007. During the study period, the pack was initially composed of 20 individuals: 10 males and 10 females, 9 adults (defined as older than two years) and 11 youngsters (younger than 2 years: 2 females born in the previous year, 6 females and 2 males of approximately 2 years old). Before the data collection started, one breeding female was removed because badly injured by the other breeding female during the reproductive season. So the study was conducted on 19 individuals. The number of individuals decreased to 14 by the end of the study period, because 4 wolves (2 adults, 2 youngsters) were removed from the pack and sold to another zoo. And finally an adult male was removed because of continuous mobbing episodes from the whole pack.

The pack was usually kept in two linked enclosures (for a total of 3000 m^2^) located in a naturally hilly area equipped with trees, branches and dens. From November to March the animals were allowed to use a larger space (for al total of 7000 m^2^) since a third linked enclosure was available. This routine is repeated every year, because the extra enclosure houses bears, that at this time hibernate and therefore are not present. The rate of aggression was actually smaller when the wolves were in the smaller enclosure (hourly rate larger enclosure = 14.4 vs. smaller enclosure = 5.02), and this is most probably due to the breeding period ending. During the study period the alpha female did not get pregnant (probably due to her old age) but three females gave birth to puppies. These puppies were killed by the alpha female and male or removed by the keepers. The animals were fed with pieces of meat, which were put on a 2 m^2^ table, 4 or 5 times every week in the early afternoon. Water was available ad libitum. No stereotypic or aberrant behaviours characterized the study group. The animals were used to the presence of tourists around the enclosure and did not show fear-related behaviour in their presence. For a detailed description of the subjects see Table 1 in Cafazzo et al. [39].

**Table 1:** Dyadic affiliative score.

	Mac	Vik	Mas	Sec	Sfr	Due	Zam	Unb	Tag	Lac	Can	Sos	Mul	Vol	Nor	Muc	Sto	Pro	Hus
Mac		1.16	0.55	0.60	0.31	0.20	0.25	0.41	0.28	0.39	0.26	0.24	0.31	0.49	0.10	0.48	0.06	0.02	0.26
Vik			0.03	0.32	0.15	0.12	0.20	0.49	0.10	0.62	0.18	0.34	0.20	0.44	0.23	0.32	0.00	0.02	0.23
Mas				0.10	0.23	0.13	0.13	0.68	0.26	0.97	0.06	0.06	0.03	0.87	0.00	0.32	0.03	0.00	0.13
Sec					0.29	0.48	0.33	0.27	0.34	0.39	0.20	0.31	0.42	0.58	0.19	0.49	0.06	0.05	0.35
Sfr						0.14	0.21	1.21	0.29	0.71	0.22	0.12	0.33	0.65	0.16	0.34	0.06	0.06	0.06
Due							0.48	0.32	0.19	0.19	0.16	0.24	0.21	0.27	0.00	0.24	0.06	0.03	0.16
Zam								0.15	0.53	0.30	0.33	0.36	0.25	0.48	0.03	0.33	0.00	0.20	0.16
Unb									0.31	0.49	0.28	0.25	0.34	2.32	0.05	0.46	0.13	0.11	0.06
Tag										0.32	0.23	0.10	0.18	0.36	0.13	0.25	0.10	0.02	0.06
Lac											0.26	0.16	0.28	0.48	0.52	0.57	0.29	0.03	0.10
Can												0.20	0.14	0.27	0.03	0.41	0.10	0.11	0.06
Sos													0.11	0.12	0.10	0.20	0.00	0.09	0.77
Mul														0.21	0.00	0.13	0.06	0.12	0.03
Vol															0.32	0.55	0.26	0.08	0.10
Nor																0.23	0.00	0.03	0.03
Muc																	0.16	0.14	0.55
Sto																		0.00	0.00
Pro																			0.00

### Data collection

Data were collected from the end of January to the end of May 2014. The pack was observed 6 days per week for 2–3 hours a day, either in the morning or the afternoon (the afternoon period included feeding time). Before commencing systematic data collection, the observer (M.L.) underwent a previous 7 month training period on wolf behaviour whilst collecting data at the Wolf Science Centre (Ernstrbunn, Austria). Moreover the observer underwent a 50 h training period to become skilled in the identification of the individuals within the pack. Although Arctic wolves are very similar to each other the observer was able to clearly recognize and distinguish all specific pack members based on some differences related to their body size, shades of grey in their fur, shapes of the muzzles and scars.

The all occurrences method was used, when an aggressive event was observed it was video-recorded for later analyses. If it wasn't possible to record the entire encounter we nevertheless made a note of: (1) the identity of the opponents, (2) the context (presence or absence of food), (3) the aggressive behavioural patterns (S1 Table), and (4) the outcome of the conflict based on the immediate answer of the victim (decided: the victim performed a submissive behaviour; undecided: the victim acted neutral, dominant or aggressive). Agonistic interactions were categorized according to two stages of aggressive intensity: stage 1 –aggressions without physical contact (threat, chase, jaw spar and snap) and stage 2 –aggressions with physical contact (attack, knock down, stand over aggressive, pin, fight, and bite) (S1 Table). An aggressive encounter was considered a new event if it occurred after one minute from the previous one and if no affiliative interaction involving the victim occurred in the meantime [57,58].

After the end of the aggressive encounter we filmed the victim as the focal individual for a 10-min post-conflict period (PC). Control observations (matched controls-MC) took place the next possible day at the same time as the original PC, on the same focal animal, in the absence of agonistic interactions during the 10 min before the beginning of the MC [38,45,59–61]. Videotapes of the PC and MC observations were loaded on a computer and analysed using the software Solomon Coder^®^ (András Péter).

Background information on the social relationships among individuals in the pack was collected using the “all occurrences” method [62] (see General Observation below). In addition to aggressive interactions, all observed affiliative, dominance, and submissive interactions both in the presence and absence of food were recorded. Videos of the PC/MC were coded looking at the affiliative and agonistic (dominance, submissive and aggressive) interactions between the victim and the aggressor and bystanders, as well as the victim’s stress-related behaviours. All behaviours are listed and described in the ethogram (S1 Table).

### Behavioural measures and analyses

#### General observations

In order to characterize the social relationship of each dyad within the pack we calculated: a) an affiliation score and b) the relative rank position of each individual. The dyadic affiliative score was calculated by adding all affiliative behaviours of A towards B and B towards A, normalized by observation time (i.e. the number of days in which both animals were present in the enclosure—Table 1).

All dominance and submissive behaviours where reported in separate matrices in order to calculate the rank relationships between individuals (S2 and S3 Tables). Linearity, DCI (directional consistency index) and I&SI rank orders were calculated separately for each matrix using Matman 1.1 (10.000 randomizations; Noldus Information Technology, Wageningen, The Netherlands) [63].

#### PC/MC observations

Alongside the general observations of the social interactions amongst the members of the wolf pack, we analyzed the occurrence, latency and frequency of affiliative, agonistic, and stress-related behaviours that occurred during PC and MC periods (S1 Table). Indeed, behavioural indicators of stress have not been previously investigated in wolves. However, self-directed behaviours (e.g. scratching and autogrooming) have been considered by some studies to be good indicators of anxiety (at least in primate species) [64,65] and a number of studies on domestic dogs have suggested that as well as self-directed behaviours also yawning and lips licking may be associated with stress [66,67,68]. These behaviours were therefore included in the current study as potential indicators of anxiety.

Aggressive interactions were categorized in: *re-aggression by the aggressor* towards the victim *counter-aggression* by the victim to the aggressor, *redirected aggression* by the victim towards a bystander, and *aggressive intervention* by a third-party towards the victim.

Conflicts were also categorized depending on the context in which they occurred, i.e. whether they occurred in the presence or absence of food (food vs. non-food context).

To assess whether ‘reconciliation’ occurred in wolves we analyzed whether friendly post-conflict interactions between former opponents occurred only or earlier in the PC than the corresponding MC period [59]. To work out standardized indices of the tendency for friendly interactions between former opponents, we adopted the method developed by Veenema et al. [69]. Hence, we determined the number of attracted (A), dispersed (D) and neutral (N) pairs over all PC–MC pairs. In attracted pairs, affiliative contacts occurred earlier (or only) in the PC than in the MC observation periods; in dispersed pairs affiliative contacts occurred earlier in the MC than in the PC (or they did not occur at all in the PC). In neutral pairs, affiliative contacts occurred during the same minute in the PC and the MC, or no contact occurred in either the PC or the MC. To avoid coding the same incident twice, for each individual we used only PC–MC pairs in which that individual was the focal animal.

Wilcoxon matched-pair signed-ranks test (corrected for ties) [70] was used to assess difference between attracted and dispersed pairs, and to assess whether, where a post-conflict affiliation interaction occurred it did so sooner than in the matched control. We further noted whether it was the victim or the aggressor that initiated the first post-conflict friendly interaction and a Mann-Whitney test was used to compare the frequency of victim-initiated vs. aggressor-initiated friendly interaction.

Following Veenema et al. [69], to calculate each individual’s corrected conciliatory tendency (CCT), we followed the formula: ‘attracted minus dispersed pairs divided by the total number of PC–MC pairs’. Individuals involved in less than 3 conflicts as victim were excluded from this calculation. Individual CCTs were then used to determine the mean group CCT. All nonparametric tests (two-tailed) were conducted in SPSS v.19. Probability level for rejection of the null hypothesis was set at 0.05.

#### Test Models

To test our main hypotheses we used Generalized Linear Mixed-effects Models (GLMMs) and accounted for possible over-dispersion in the data, when necessary, running glmmPQLs (R Development Core Team 2016). Since individuals were present in multiple dyads and each dyad could occur more than once (when involved in more than one conflict), we included dyad and individual as a random factors in the models to avoid pseudo-replication.

We used a backward stepwise reduction procedure based on p-values [71] to remove non-significant terms [72]. All model analyses were performed using R v3.2.5. We implemented generalized mixed-effects models using the “glmer” function in the “lme4” package [73]; the “glmmPQL” function was fitted using the “nlme” [74] and “MASS” [75] packages [76].

#### Model 1

To assess which factors may affect the likelihood of reconciliation occurring we ran a generalized mixed-effect model (GLMM with a binomial distribution) with the occurrence of reconciliation (yes/no) as the response factor and conflict intensity (high or low), context (food vs. non-food), opponent sex combination (male-male, male-female, female-female), rank position of the victim in relation to the aggressor (subordinate vs. dominant), the affiliation score of each dyad and the season (mating season vs. non-mating season) as independent factors.

#### Model 2

The aim of the current model was to test whether conflicts between individuals with a closer social bond (higher affiliation score) resulted in the victim displaying more stress-related behaviours. To evaluate this question, we calculated the frequency of stress related behaviours as the sum of all stress signal events occurring in PCs in which no reconciliation (or any other affiliative interaction between the victim and a bystander) occurred (n = 77) and all stress signal events occurring before the reconciliation event (or before a third party affiliation event if this occurred before reconciliation) in the PCs in which reconciliation did in fact occur (n = 30). This measure was normalized by observation time. A GLMM with Poisson distribution was used to assess whether the affiliation score between the victim and the aggressor affected the frequency of stress signals. We included intensity of the aggressive conflict, context (food and non-food), sex combination, and rank relationship between aggressor and opponent as variables potentially affecting the manifestation of stress signals after the conflict.

#### Model 3

To examine the ‘reconciliatory function’ of post-conflict affiliative interactions and hence whether such interactions decrease the likelihood of renewed aggression between former opponents, we ran a generalized mixed-effect model (GLMM with a binomial distribution) with the occurrence of renewed aggression (yes/no) as dependent variable, and the prior occurrence of a post-conflict affiliative interaction between a) the opponents and b) the victim with a bystander (to control for the potential effect of bystander affiliation on the likelihood of renewed aggression between former opponents), intensity of aggression, context (food and non-food), sex combination, rank position of the victim in relation to the aggressor (subordinate vs. dominant) and dyadic affiliation score and as independent variables.

#### Model 4

To investigate the effect of post-conflict affiliation on aggression directed from the victim to a third party (redirected aggression), we ran a generalized mixed-effect model (GLMM with a binomial distribution) with the occurrence of redirected aggression (yes/no) as dependent variable, and the prior occurrence of a post-conflict affiliative between a) the opponents and b) the victim with a bystander (to control for the potential effect of bystander affiliation on the likelihood of renewed aggression between former opponents), intensity of aggression, context (food and non-food), sex combination, rank position of the victim in relation to the aggressor (subordinate vs. dominant) and dyadic affiliation score as independent variables.

#### Model 5

Furthermore, given the relative frequency of the occurrence of aggressive intervention by a third-party towards the victim, we assessed whether the occurrence of post-conflict affiliative interaction between the victim and the aggressor may function as a way to reduce the likelihood of victim-directed bystander aggression. Hence a GLMM (with binomial distribution) was run with the occurrence of victim-direct bystander aggression (yes/no) as dependent variable, and prior occurrence of a post-conflict affiliative interaction between a) the opponents and b) the victim with a bystander (to control for the potential effect of bystander affiliation on the likelihood of renewed aggression between former opponents), intensity of aggression, context (food and non-food) and rank position of the victim in relation to the aggressor (subordinate vs. dominant) as independent variables.

#### Model 6

To investigate the effect of post-conflict affiliative interaction between the victim and the aggressor on post-conflict anxiety, we calculated the frequency of stress behaviours occurring in PCs in which no such reconciliation (or any other affiliative interaction between the victim and a bystander) occurred (n = 77) and the frequency of stress behaviours occurring after the reconciliation event (but before a potential third party interaction event) in the PC in which reconciliation did in fact occur (n = 30, these are all the cases in which reconciliation occurred either before or in the absence of any affiliative event by a third party. Cases in which a third party preceded the reconciliation event were excluded from this analysis, since it was otherwise impossible to disentangle whether it was the third party affiliation event or the reconciliation event that affected the stress behaviours of the animals). Both measures were normalized for observation time. A GLMM with Poisson distribution was used to assess whether the prior occurrence of reconciliation affected the frequency of stress signals. We included intensity of the aggressive conflict, context (food and non-food), sex combination, affiliation score and rank relationship between aggressor and opponent as variables potentially affecting the manifestation of stress signals after the conflict.

## Results

### General observations

The affiliation score amongst pack members varied from a minimum of 0 to a maximum of 1.16 (Table 1). Furthermore, we could observe the presence of a linear hierarchy based on dominant behaviours (h’ = 0.58, p = 0.0001, DCI = 0.97) and one based on submissive behaviours performed amongst the wolves in the pack (h’ = 0.56, p = 0.0001, DCI = 0.97) [39]. Since the matrix of dominant behaviours generated two inconsistencies and the matrix of submissive behaviours showed no inconsistencies, we considered submissive behaviours as most reliable indicator of linear dominance relationships. Based on these data individuals in the pack were ordered from the highest to the lowest ranking animal, hence for each dyad involved in a conflict we were able to determine if the victim was subordinate or dominant to the aggressor. For further details see Cafazzo et al. [39].

### PC/MC observations

In 154 hours of observations a total of 585 aggressions with physical contact were recorded (3.8 aggressions per observation hour), and 386 aggressions with no physical contact (2.5 per observation hour). A total of 133 PC/MC pairs were recorded involving a total of 19 subjects (i.e. all animals in the pack) as victim. The number of aggressions per individuals ranged from 1 to 35, mean conflicts per focal was 7. Of the total 133 PC episodes, 14 were undecided and 115 were decided (the outcome of 4 conflicts was not clear to the observer); 46 were of low and 86 of high intensity (the intensity of one conflict was not clear); 52 occurred in the food context and 81 outside of it.

#### Post-conflict friendly interactions: Do they occur?

Considering reconciliation at the group level, we found a significant difference between attracted and dispersed pairs (attracted pairs > dispersed pairs, Wilcoxon’s Z = 3.6, ties = 0, n = 17, p < 0.0003). The mean CCT of all focal individuals was 46.87% (Table 2).

**Table 2:** Corrected conciliatory tendencies (CCT), number of attracted, dispersed and neutral pairs for each victim. Subjects are listed following the rank order.

Focal /Victim	attracted	dispersed	neutral	Total	CCT%
**Secondo**	2	0	1	3	66.67
**Sfregiato**	1	0	7	8	12.5
**Zampa**	3	0	2	5	60
**Due**	2	0	1	3	66.67
*Unobeta*	4	0	11	15	26.67
*Volpe*	7	1	6	14	42.86
*Lacrima*	11	1	6	18	55.57
*Cane*	4	0	2	6	66.67
**Taglio**	2	0	2	4	50
**Musolungo**	2	0	6	8	25
*Musocorto*	*10*	*3*	*22*	*35*	*20*
*Sosia*	*2*	*0*	*2*	*4*	*50*
**Storto**	2	0	1	3	66.67
**Procione**	0	0	2	2	0
**Total**	**52**	**5**	**69**		
**Group CCT% SEM**					**46.87** **5.46**

Bold type: males; italic type: females

**Fig 1:**
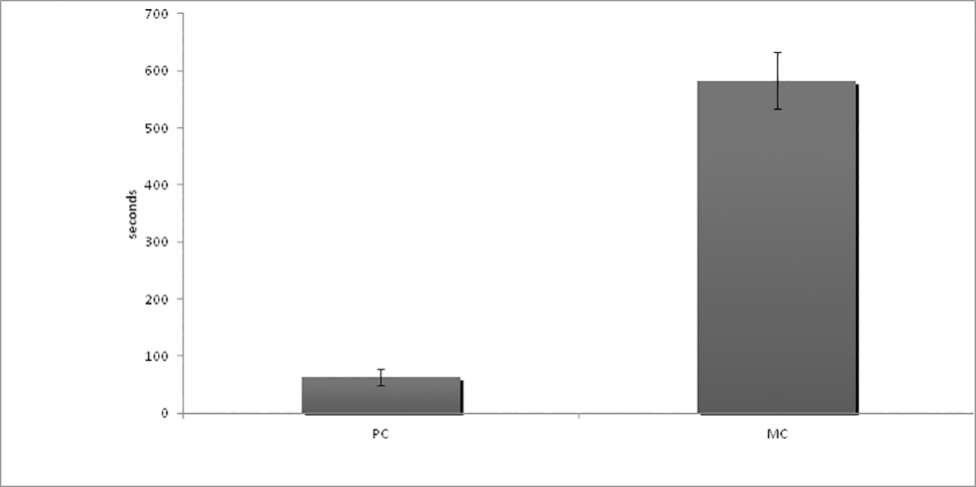
Mean latency to the first affiliative interaction in PC and MC periods.

Furthermore, where an affiliation interaction occurred in the PC period, it did so sooner than in the MC period (Wilcoxon: N = 56, z = 6.5, p<0.001) (Fig 1).

**Fig 2:**
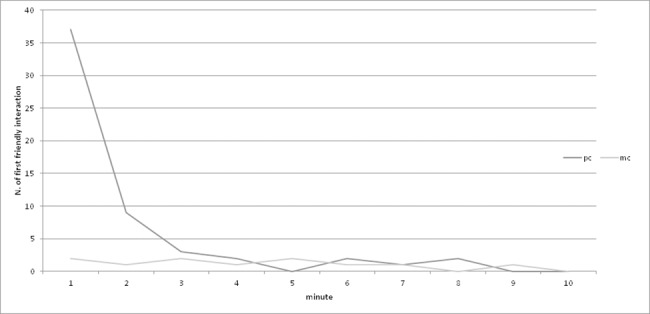
Temporal distribution of first affiliative interactions in PC and MC periods.

The majority, i.e. 78.56% (44 of 56) of first friendly contacts occurred within 2 minutes of the conflict compared to only 33.33% (4 of 12) in the matched control period (Fig 2). There was a significant difference in the number of affiliative behaviours occurring in PC vs. MC periods in the first minute (χ^2^ = 28.66, p < 0.001), only a tendency to a significant difference in the second minute (χ^2^ = 3.13, p = 0.08) and no significant difference in in the third (χ^2^ = 0.5, p = 0.48).

#### Who is more likely to initiate a post-conflict friendly interaction and what behaviours are used more often?

When a post-conflict friendly interaction occurred between opponents it was more likely to have been initiated by the victim (38) than the aggressor (18) (Mann-Whitney: N = 56, z = 2.18, p = 0.03).

The most frequent affiliative behaviours being exhibited as the first friendly interaction between opponents involved either some form of body contact (54% i.e. 9 body-rubbing, 2 inspection, 1 body contact, 1 groom, 3 social sniff, 2 nose touch, 1 paw touch, 11 muzzle licking) or a search for proximity in a friendly manner (38% i.e. 7 approach friendly, 11 stand friendly, 3 play invitations).

#### Patterns of aggressions during post-conflict observations

The analysis of the post-conflict aggressive interactions recorded revealed that the percentages of PC samples in which re-aggression from the aggressor to the victim, counter aggression by the victim to the aggressor and redirected aggression by the victim to a third party were relatively low (i.e. 13.5%, 1.5%, 9%, respectively). In contrast, third party aggression towards the victim occurred in a fourth of PC periods (25.5%).

#### What factors affect the occurrence of post-conflict friendly interactions (Model 1)?

**Fig 3:**
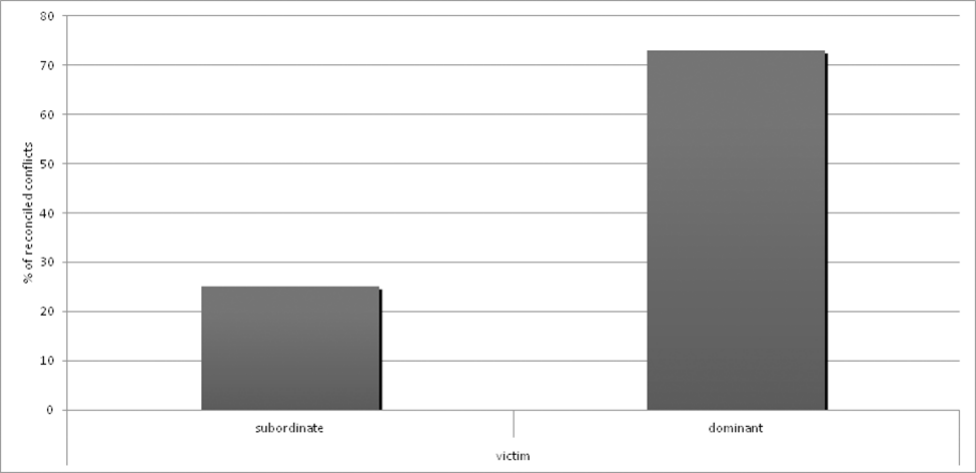
Effect of rank relationship between opponents on the occurrence of post-conflict affiliative interactions.

Following a backward stepwise reduction, the final GLMM, showed an effect of rank relationship between opponents (glmm: χ^2^ = 6.63, p = 0.01) and context (glmm: χ^2^ = 4.30, p = 0.04). With the likelihood of post-conflict friendly interactions occurring more often when the victim was dominant and the aggressor subordinate (Fig 3) and when the conflict happened outside the food context.

Post-conflict affiliative interactions occurred more often when the victim was dominant and the aggressor subordinate

In fact of the total conflicts recorded, 91.72% (n = 122) were directed from a dominant individual towards a subordinate. Of these 38.52% (n = 47) were followed by a friendly contact, in most cases (63.83%) initiated by the victim (n = 30). When the aggression did occur from the subordinate towards the dominant (8.27%, n = 11), in 81.81% (n = 9) of cases the conflict was followed by a friendly interaction, and in 8 cases (88.89%) the victim initiated these.

As regards the context of the aggression, of the 52 conflicts occurring when food was available 32.69% (n = 17) were followed by a friendly interaction, whereas of the 81 conflicts occurring in the non-food context 48.15% (n = 39) were followed by a friendly interaction.

Intensity of the conflict (glmm: χ^2^ = 1.61, p = 0.20), affiliation score (glmm: χ^2^ = 0.81, p = 0.37), season (glmm: χ^2^ = 0.38, p = 0.54), and sex combination (glmm: χ^2^ = -0.03, p = 0.99) between opponents had no effect on the likelihood of a friendly interaction occurring.

#### What factors affect the victim’s rate of stress related behaviours (Model 2)?

A total of 943 stress-related behaviours were observed in the PCs and 360 were observed in the MCs. Self-directed behaviours (i.e. scratching and auto-grooming) were quite uncommon representing respectively the 0.74% and 4.77% of stress-related behaviours in the PCs and 0.83% and 7.22% of total behaviours in the MCs. Body shaking, yawning and lips licking represented respectively 8.8%, 9.86% and 75.82% of stress-related behaviours in PCs and 14.44%, 6.39% and 71.11% in MCs.

Stress-related behaviours in the victim occurred significantly more often during post-conflict periods than during the corresponding matched-control periods (Wilcoxon: mean PC = 7.2 vs. MC = 2.7; z = 7.24, N = 133, p <0.001), indicating that conflicts are stressful for victims. Following a backward stepwise reduction, we found that stress signals displayed before any reconciliation (or bystander affiliation) event or when neither reconciliation nor bystander affiliation occurred, were more frequently displayed by victims after conflicts characterised by high intensity aggressive behaviours (glm: χ^2^ = 2.73, p = 0.01) and occurring during non-feeding interactions (glm: χ^2^ = 3.52, p = 0.001). The affiliation score between victim and aggressor (glm: χ^2^ = 0.55, p = 0.59), rank relationship (glm: χ^2^ = 0.77, p = 0.45) and sex combination (glmm: χ^2^ = 0.49, 0.62) between opponents had no effect on the victim’s rate of stress signals.

#### Reconciliatory function of Post-conflict friendly interactions: Do they reduce the likelihood of re- aggression (Model 3), redirected aggression (Model 4) and/or of third-party aggression towards the victim (Model 5)?

Of the 18 cases of *re-aggression*, 33.33% (n = 6) occurred after the occurrence of a post-conflict friendly interaction and 66.67% (n = 12) occurred in the absence of such a behaviour. Following a backward stepwise reduction (Model 3), the final GLMM showed that the occurrence of a friendly interaction between aggressor and victim reduced the likelihood of re-aggression occurring (glmm: χ^2^ = 4.79, p = 0.03). Neither context in which the conflict occurred (glmm: χ^2^ = 0.02, p = 0.96), conflict intensity (glmm: χ^2^ = 0.03, p = 0.87), affiliative relationship between victim and opponent (glmm: χ^2^ = 1.62, p = 0.20), rank relationship (glmm: χ^2^ = 0.39, p = 0.53), sex combination (glmm: χ^2^ = 3.18, p = 0.20) of opponents nor the occurrence of a bystander affiliation (glmm: χ^2^ = 0.79, p = 0.38) affected the likelihood of re-aggression.

Of the 12 cases of *redirected aggression* (n = 12), 8.33% (n = 1) occurred after the occurrence of a post-conflict friendly interaction and 91.67% (n = 11) occurred in the absence or before, such a behaviour. Indeed, following model reduction (Model 4), the occurrence of a friendly interaction reduced the likelihood of redirected aggression occurring (glmm: χ^2^ = 8.34, p = 0.004). The occurrence of bystander affiliation (glmm: χ^2^ = 1.69, p = 0.19), the intensity of the conflict (glmm: χ^2^ = 0.20, p = 0.66), and the context in which it occurred (glmm: χ^2^ = 0.04, p = 0.83) did not affect the likelihood of redirected aggression occurring. Nor did any sex combination (glmm: χ^2^ = 3.81, p = 0.15), rank relationship (glmm: χ^2^ = 0.00, p = 0.99) and affiliative score (glmm: χ^2^ = 0.88, p = 0.35) between opponents.

Of the 34 cases of *third party aggression* towards the victim, 29.41% (n = 10) occurred after the occurrence of a post-conflict friendly interaction and 70.59% (n = 24) occurred in the absence of such a behaviour. Following a backward stepwise reduction (Model 5), the occurrence of post-conflict friendly interaction between the opponents (glmm: χ^2^ = 2.17, p = 0.14) as well as the occurrence of a bystander affiliation (glmm: χ^2^ = 2.17, p = 0.14) had no effect on the likelihood of a third party aggression on the victim occurring. Nor did the sex combination (glmm: χ^2^ = 1.70, p = 0.43), affiliation score (glmm: χ2 = 0.22, p = 0.83) and rank relationship (glmm: χ^2^ = 2.81, p = 0.09) between the opponents. However, the conflict intensity (glmm: χ^2^ = 11.53, p = 0.0007) and the context in which it occurred (glmm: χ^2^ = 7.15, p = 0.008) affected the likelihood of a third party aggression on the victim. In 30.23% (26 of 86) of cases in which there was an initial aggression involving physical contact a bystander also showed aggression towards the victim, whereas this occurred in only 17.39% (8 of 46) of cases where the initial aggression did not involve physical contact. As regards the context, of the 52 conflicts occurring during feeding, 21.15% (n = 11) were followed by a third party aggression on the victim, whereas of the 81 conflicts in the non-feeding context, 28.39% (n = 23) were followed by a third party aggression on the victim.

#### Reconciliatory function of Post-conflict friendly interactions: Do they reduce stress-related behaviours in the victim (Model 6)?

Following a backward stepwise reduction, friendly interaction between opponents (glm: χ^2^ = 0.20, p = 0.65), intensity of the aggression (glm: χ^2^ = 0.55, p = 0.46), rank relationship (glm: χ^2^ = 1.22, p = 0.23), sex combination (glmm: χ^2^ = 1.33, 0.51) as well affiliation score (glm: χ^2^ = 0.53, p = 0.47) between opponents did not affect the frequency of stress behaviours. There was a trend towards more stress behaviours being shown outside the feeding context (glmm: χ^2^ = 0.89, p = 0.05).

## Discussion

Our results confirm the existence of post conflict affiliative interactions (POAs) between former opponents of a conflict in a captive family group of arctic wolves. In fact, we found that the rate of POAs was higher after a conflict than during a control situation when no aggressive events occurred. Moreover the POAs tended to occur soon after the conflict concentrating in the first two minutes, indicating that their occurrence was dependent on the preceding conflict. The shorter latency in performing affiliative behaviours after the conflicts than the relative controls indicates that the former opponents show higher affiliative tendency in post-conflict situations. Although captive conditions do not completely reflect field situations, especially as regards the opportunity for dispersion and cooperation (e.g. group hunting and territorial defence), the mean conciliatory tendency of our pack (CT = 46.87%), was comparable to the one observed in two family groups of free ranging Canadian Timber wolves (CT = 44.1% [45]) and just a little under the one observed in a captive pack of grey wolves (CT = 53.2% [38]). In general, considering conciliatory tendencies reported in other species vary from 0 to 50% (hyenas [77], chimpanzees [8,78,79], bonobo [80], baboons [5,11], ravens [14], common marmosets [57], tamarins [58], lemurs [81,82], meerkats [83]) wolves appear to be in the higher ranges, suggesting these behaviours may play an important role in their social lives.

However, most of the predictions of the ‘integrated hypothesis’ were not supported by our current results. Firstly, despite stress-related behaviours occurring more in PC than MC periods, and more after high vs. low intensity aggressions (hence suggesting that they were in fact a measure of the animals’ level of anxiety), they were not more likely to occur after conflicts between wolves with a stronger social bond and POAs did not reduce these in the victims. These results suggest that the victim’s state of stress may be caused by factors independent of the opponents’ relationship. We observed that after a conflict, victims were targets of aggression from bystanders in 25.5% of cases and POAs did not decrease the likelihood of their occurrence. Therefore, it is possible that victim’s stress level was associated with the perceived high risk of receiving a new aggression from a third party not involved in the original conflict. This conclusion is further supported by our findings that a third party aggression to the victim was more likely to occur after conflicts characterised by high intensity aggression and occurring in the non-feeding context, two factors which we found were also related to higher levels of stress in the victim. Another possibility is that the victim’s stress-signals decrease only if the post-conflict affiliative interaction is in fact reciprocated (the receiver of the POA answers with an affiliative behaviour instead of acting in a neutral manner), indicating that the bond has indeed been repaired. This aspect has so far received almost no attention in reconciliation studies, hence there is little scope for comparison, however it is interesting to note that in the current study only 1.2% of POAs were in fact reciprocated.

Secondly, in contrast to the ‘integrated hypothesis’, POAs were not more likely to occur between opponents with higher relationship quality. These results are in line with those from a study of reconciliation in captive grey wolves [38] as well as studies in other species that depend on cooperative activities such as group hunting (e.g. hyenas [77]) or cooperative breeding (callitrichids [84], lemurs [81,82]; meerkats [83]) (although different from results from other species relying like wolves on pair-bonding e.g. ravens [14]). In cooperative species, such as wolves, the potential loss of benefits due to the disruption of cooperation caused by the conflict is expected to serve as an incentive to pursue reconciliation [85]. This suggests that a cooperation index (i.e. cooperation in raising litters or during hunting), instead of the affiliation score may have been a better measure to evaluate the dyadic relationships and we could expect dyads with a higher cooperation index to reconcile more. Unfortunately we were not able to collect such information in this study. However, interestingly Palagi et al. [38] found that POA occurred more frequently among those individuals sharing higher levels of coalitionary support during conflicts, further suggesting that a measure of a dyads cooperative interaction may be a better indication than affiliation *per se*.

Results are also consistent with studies of reconciliation in species whose social organization is mostly represented by a high degree of relatedness between group members (e.g. ring-tailed lemurs [81]). Taken together, results support the hypothesis that in wolves it may be in the interest of all dyads to restore disrupted relationships [38,45,86], and that relationship quality as a mediating factor in the occurrence of POAs may be linked to the degree of relatedness in a species’ social organization and their reliance on cooperative behaviours [45,87].

Two main factors played a role in the likelihood of a POA occurring: the dominance relationship between opponents and the context in which the conflict occurred. In the rare cases (8.27% of conflicts) in which the victim was dominant to the aggressor, POAs occurred at a high rate 81.81%. Whereas POAs occurred only on 38.52% of occasions in which the victim was subordinate to the aggressor. Hence, although relationship quality did not affect the occurrence of POAs, the role during the conflict and the dominance relationship between opponents contributed to the occurrence of such behaviours. When victims were dominant to the aggressor reconciliation was more likely to occur. This is probably due to the fact that the potential risk of being re-aggressed is low, whereas subordinate individuals have to take into account the likelihood of being re-aggressed when carrying out a reconciliation attempt. Furthermore, consistent with previous finding in other species (e.g. [82,88–91]), POAs turned out to occur more often in the non-feeding context than during feeding sessions. As a form of direct competition, contests over food can be particularly risky [92] and this may limit the likelihood of post-conflict reunions occurring [93]. Furthermore, during feeding, affiliative interactions may be unlikely because animals are busy eating.

Finally, our results showed that POAs potentially serve an important function in wolf packs, in that they are effective in reducing the occurrence of renewed aggressions between opponents and redirected aggressions from victims towards bystanders. Such results are in accordance with a number of previous studies in other species (hyenas [77], macaques [2,3,7,43], ravens [14], lemurs [82]), but contrast with the only other study looking at this aspect in wolves. Indeed Baan et al. [45] did not find support for renewed aggression being less likely after situations in which post-conflict affiliations had occurred. However, the low number of conflicts recorded in the wild (only 34 conflicts [45]) may account for such contrasting results.

The pattern of results regarding the initiator of the POAs, is also consistent with the possibility that the main function of these behaviours for wolves is to reduce the risk of further aggressions occurring. In fact, we found that the victim engaged in POAs more often than the aggressor (68% of cases), consistent with the fact that its actions can reduce the probability of receiving a re-aggression (which are quite common, occurring in 13.5% of cases). On the contrary the aggressor, who rarely received counter aggressions from the victim (only in 1.5% of cases), engaged in POAs in only 32% of cases. Hence, the victim’s decision to engage in POAs appears to be affected by striking a balance between the risk of receiving a new aggression (relating also to the dominance status of the partner) and the benefits of succeeding in the attempt, thereby reducing the probability of being re-attacked.

A possible, though not exclusive, hypothesis is that POAs in wolves have been selected for, as a mechanism to avoid conflict escalation, which could lead to social disruption and hence jeopardize cooperative activities. The high relatedness among individuals in the pack may reduce the importance of ‘relationship quality’ as a mediating factor of POAs, although dominance relationships, being directly linked to risks of further conflicts, do play an important role.

A number of limitations of the current study need to be considered. Firstly, as always with studies in captivity, results regarding the function of specific behaviours would benefit from confirmation from studies in the wild. Observational studies of wild wolves, are a challenge, nevertheless, it is our hope that the current methodologies may at least pave the way for such studies with wild animals. More specifically, regarding wolves, a long-standing debate has centred around the possibility that wolves’ hierarchical organization observed in captive populations does not reflect, the hierarchical organization observed in wild packs [40,94]. This debate will also only be resolved with careful behavioural observations of wild populations, which are currently missing. Nevertheless, it is important to note, that an in depth exploration of the social structure of the pack under investigation was carried out and is reported elsewhere [39]. Another, related issue, is that levels of aggression have been observed to be higher in captive compared to wild packs [95], and considering that the current pack had undergone the disruptive event of loosing one of its breeding females and other four members and the death/removal of the year’s pups, conflicts may have been particularly frequent at the time of the data collection. However, it is interesting to note that regardless of the frequency of aggressive interactions, reconciliation rates in the current study were actually very similar to those in the only study carried out on wild wolves [45]. Overall, current results regarding the function of reconciliation, should be viewed with a measure of caution, and, as always, will benefit from replication in other captive populations, and even more importantly, wild packs of wolves.

## Conclusion

In conclusion, our results suggest that a possible function of post-conflict opponent affiliative interactions in wolves is to reduce the risk of further aggressions occurring. Our findings do not support the *Integrated Hypothesis* that has been proposed for primates, suggesting that the same behavioural patterns may play a different role in species that show a different social structure (higher relatedness) and that are more reliant on cooperative activities (cooperative breeders and hunters). A broader comparative approach taking into account differences in social structure and ecology between species will help to further elucidate the potential function of post-conflict affiliative interactions.

## Supporting information

S1 Table Ethogram. List and description of the behaviours recorded.
(PDF)

S2 Table Dominant behaviours. Matrix of dominant behaviours recorded.
(PDF)

S3 Table Submissive behaviours. Matrix of submissive behaviours recorded.
(PDF)
