# Supplementary material for: Post-conflict opponent affiliation reduces victim re-aggression in a family group of captive arctic wolves (Canis lupus arctos)
Source: PLoS One. 2017 Nov 6;12(11):e0187450. doi: 10.1371/journal.pone.0187450 (PMC5673216; doi:10.1371/journal.pone.0187450)
Supplement: S1 Table — List and description of the behaviours recorded. (PDF) [file pone.0187450.s001.pdf]

S1 Table. Ethogram.

Category	Behaviours	Description
**Affiliative behaviours**		
	Grooming	To nip, lick or scratch the fur or skin occasionally the neck of the receiver's
	Inspection	To interact friendly, stand next to each other, rubbing against each other side by side, smelling each other, putting heads together and licking, sniffing and so on
	Play invitation	Two or more subjects engage in motor patterns such as bite, chase, run around one another, kick, jump and maybe snap or bite without enough pressure to cause injury. These patterns can be typical of 'serious' functional contexts (e.g. agonistic, anti-predatory) but in a different manner. In fact, playful behaviours are often exaggerated, reordered, incomplete, brief, repeated, varied in sequence and inhibited. A play bout is considered over after 30 seconds of non-play.
	Play	Two or more subjects engage in motor patterns such as bite, chase, run around one another, kick, jump and maybe snap or bite without enough pressure to cause injury. A play bout is considered over after 30 seconds of non-play
	Lie friendly	To lie on the back, tail-wagging, maybe kicking with the foreleg against or toward another subject sometimes with open mouth
	Stand friendly	The subject stands with tail horizontal to or below the plane of the back, wagging it, ears pointed forward, while another is approaching it or orienting/looking towards it
	Body contact	Two subjects stay (for at least 10 s) with at least a part of their bodies in contact and in a relaxed position
	Social sniff	To sniff another's body part except its anogenital area
	Body rubbing	To rub ones body against any part of the receiver's
	Approach friendly	To approach another subject within one body length remaining within that distance for at least 5 seconds. The approach is characterized by the subject holding the tail perpendicular to or below the plane of the back and wagging it
	Nose touch	Brief nose touch by one wolf to the face or body of another wolf; no tail wag, ears may be flattened
	Muzzle licking	To lick the other's lips and nose. The tail is relaxed and below the plane of the back
**Dominance behaviours**		
	Stand tall	Subject traightens up to full height, with a rigid posture and tail, may include raised hackles, ears erect and tail perpendicular or above the back
	Stand over	To stand over another's body, with all four paws on the ground and the tail above the plane of the back. The receiver may have either the whole body or just the forepaws under the actors' belly/side
	Paw on	To place one or both forepaws on the other's back
	Ride up	To mount another one from behind or from the side, exhibiting a thrusting motion
	Head on	The subject approaches another's shoulder/back with the tail above the plane of the back and puts its head on it. Most of times formation looks like a capital "T"
	Muzzle bite	To grab the muzzle of another subject softly

Approach dominant	To approach another subject within one body length for at least 5 seconds, with the tail perpendicular or above the plane of the back and the ears erect and pointed forward

Submissive behaviours

Crouch	Lowering the head, sometimes bending the legs, arching the back, lowering the tail between the hindlegs, and avoiding eye contact
Passive submission	To lie on the back showing the stomach and holding the tail between the legs. The ears are held back and close to the head and the subject raises a hind leg for inguinal presentation
Active submission	The subject has its tail tucked between the hind legs sometimes wagging it while he is in a crouched position (with hindquarters lowered) and may attempt to paw and lick the side of actors'/aggressor's muzzle. The behaviour may include urination
Play submissive	To play with the tail between the hind legs, often running away and snapping at the other
Withdrawing	The subject withdraws from another moving away slowly in the opposite direction, displaying a submissive posture. It occurs when a subject has been threatened or attacked by another, or a fight has taken place
Flee	To run away from another with tail tucked between the legs and body ducked. It occurs when a subject has been threatened or attacked by another, or after a fight
Avoidance	In response to another reducing the distance towards it, the subject moves away displaying a submissive posture. The subject may also look at the individual he is trying to avoid
Approach submissive	To slowly approach another within one body length remaining within that distance for at least 5 seconds. The approach is characterized by a ducked posture and tail between the legs. Subject can also be moving in a wavy line and in a hesitant (stop-start) manner

Aggressive behaviours

Threat	Subject orients towards another performing one or more of the following: staring at, curling of the lips, baring of the canines, raising the hackles, snarling, growling, and barking, sometimes with the tail perpendicular or above the back
Attack	Running into or jumping onto another with tail, ears and sometimes hackles up, often with bites at the neck
Knock down	To strike another subject sharply with the chest or shoulder so that the other falls to the ground
Stand over aggressive	To overwhelm another subject, stopping on it in a stand over position with the tail above the plane of the back, growling and showing piloerection
Pin	To grab another at the neck or at the muzzle, forcing it down to the ground and holding it there
Fight	The subject and the receiver engage in reciprocal biting and aggressive physical contact
Chase	A subject runs after a conspecific, exhibiting threatening behaviours (see 'threat' above)
Jaw spar	Two subjects "fencing" with open jaws
Snapping	To snap teeth into the air, noisily
Bite	Bite a conspecific, without inhibition, with enough pressure to cause potential injury

	Submissive aggression	The subject is in a crouched posture, tail tucked in and sometimes with a paw up but shows teeth whilst having the corners of the mouth pushed forward, often accompanied by snapping, pushing the other away, and/or barking
**Self directed behaviours**		
	Autogrooming	To lick and nibble its own skin and fur
	Body shaking	To shake the body or the neck
	Yawning	To open the jaws wide without vocalising
	Lips licking	To extrude the tongue from the mouth and run it over the lips
	Scratching	To scratch any part of the body
