# Supplementary material for: Post-conflict opponent affiliation reduces victim re-aggression in a family group of captive arctic wolves (Canis lupus arctos)
Source: PLoS One. 2017 Nov 6;12(11):e0187450. doi: 10.1371/journal.pone.0187450 (PMC5673216; doi:10.1371/journal.pone.0187450)
Supplement: S2 Table — Matrix of dominant behaviours recorded. (PDF) [file pone.0187450.s002.pdf]

*	macchia	viki	maschera	secondo	sfregiato	due	zampa	uno beta	taglio	lacrima	cane	sosia	muso lungo	volpe	normale	muso corto	storto	procione	husky
macchia	*	5	17	7	15	5	3	2	7	4	3	1	5	8	2	6	2	5	4
viki	2	*	6	7	5	4	3	78	1	38	4	3	4	39	4	9	1	0	1
maschera	0	0	*	1	36	4	1	3	4	6	1	0	4	4	0	1	3	1	0
secondo	0	0	0	*	5	1	0	1	1	1	0	3	0	1	0	3	1	2	1
sfregiato	0	0	0	2	*	2	0	2	4	1	0	2	2	2	0	1	1	1	0
due	0	0	0	0	0	*	1	0	0	0	0	0	0	0	0	2	0	0	0
zampa	0	0	0	0	0	0	*	1	1	0	0	0	0	0	0	1	0	2	1
uno beta	0	0	0	0	0	0	0	*	0	7	3	2	0	28	0	3	0	0	0
taglio	0	0	0	0	1	0	0	0	*	0	2	0	0	0	3	1	2	0	0
lacrima	0	0	0	0	0	0	0	0	0	*	11	2	0	0	14	31	0	0	0
cane	0	0	0	0	0	0	0	1	1	0	*	2	1	12	0	122	0	4	0
sosia	0	0	0	0	0	0	1	0	0	0	1	*	1	0	0	8	0	1	0
muso lungo	0	0	0	0	0	0	0	0	0	0	0	0	*	1	0	0	0	0	0
volpe	0	0	0	0	0	0	0	0	0	17	0	0	0	*	7	11	0	0	0
normale	0	0	0	0	0	0	0	0	0	0	0	0	0	0	*	1	0	0	0
muso corto	0	0	0	0	0	0	0	0	0	0	0	3	0	0	0	*	0	4	0
storto	0	0	0	0	0	0	0	0	0	0	0	0	0	0	0	0	*	1	0
procione	0	0	0	0	0	0	0	0	0	0	0	0	0	0	0	0	0	*	0
husky	0	0	0	0	0	0	0	0	0	0	0	0	0	0	0	3	0	0	*
