# Supplementary material for: Post-conflict opponent affiliation reduces victim re-aggression in a family group of captive arctic wolves (Canis lupus arctos)
Source: PLoS One. 2017 Nov 6;12(11):e0187450. doi: 10.1371/journal.pone.0187450 (PMC5673216; doi:10.1371/journal.pone.0187450)
Supplement: S3 Table — Matrix of submissive behaviours recorded. (PDF) [file pone.0187450.s003.pdf]

*	macchia	viki	maschera	secondo	sfregiato	due	zampa	uno beta	taglio	lacrima	cane	sosia	muso lungo	volpe	normale	muso corto	storto	procione	husky
macchia	*	1	0	0	0	0	0	0	0	0	0	0	0	0	0	0	0	0	0
viki	0	*	0	0	0	0	0	0	0	0	0	0	0	0	0	0	0	0	0
maschera	14	1	*	0	0	0	0	0	0	0	0	0	0	0	0	0	0	0	0
secondo	14	3	4	*	2	0	0	0	0	0	0	0	0	0	0	0	0	0	0
sfregiato	4	2	13	4	*	0	0	0	0	0	0	0	0	0	0	0	0	0	0
due	10	1	3	2	1	*	0	0	0	0	0	0	0	0	0	0	0	0	0
zampa	3	0	0	0	0	0	*	0	0	0	0	0	0	0	0	0	0	0	0
uno beta	6	48	1	0	0	1	0	*	0	0	0	0	0	0	0	0	0	0	0
taglio	13	2	3	0	7	1	1	0	*	0	0	0	0	0	0	0	1	0	0
lacrima	3	27	1	2	1	0	0	1	0	*	0	0	1	9	0	0	0	0	0
cane	2	1	0	0	0	0	0	3	0	6	*	1	0	0	0	0	0	0	0
sosia	2	1	0	0	1	1	1	0	0	0	1	*	0	0	0	11	0	0	0
muso lungo	22	5	10	0	10	0	0	1	0	2	0	0	*	1	0	0	0	0	0
volpe	4	31	1	2	2	0	0	2	0	0	0	0	1	*	0	0	0	0	0
normale	1	3	0	0	0	0	0	0	0	6	0	0	0	4	*	0	0	0	0
muso corto	13	11	0	2	2	1	0	1	1	16	42	2	1	8	1	*	0	0	0
storto	4	4	3	0	1	0	0	1	4	1	0	0	0	2	2	2	*	0	0
procione	23	1	3	2	5	7	2	1	2	1	7	1	5	0	0	3	1	*	0
husky	1	2	4	0	0	0	1	0	0	1	0	0	1	0	0	0	0	1	*
